# Scratching Counteracts IL-13 Signaling by Upregulating the Decoy Receptor IL-13Rα2 in Keratinocytes

**DOI:** 10.3390/ijms20133324

**Published:** 2019-07-06

**Authors:** Dugarmaa Ulzii, Makiko Kido-Nakahara, Takeshi Nakahara, Gaku Tsuji, Kazuhisa Furue, Akiko Hashimoto-Hachiya, Masutaka Furue

**Affiliations:** 1 Department of Dermatology, Graduate School of Medical Sciences, Kyushu University, Fukuoka 812-8582, Japan; 2 Department of Dermatology, National Dermatology Center of Mongolia, Ulaanbaatar 14171, Mongolia; 3 Division of Skin Surface Sensing, Graduate School of Medical Sciences, Kyushu University, Fukuoka 812-8582, Japan; 4 Research and Clinical Center for Yusho and Dioxin, Kyushu University Hospital, Fukuoka 812-8582, Japan

**Keywords:** scratch injury, IL-13Rα2, keratinocyte, IL-13, atopic dermatitis, IL-4Rα, IL-13Rα1, involucrin

## Abstract

The vicious itch–scratch cycle is a cardinal feature of atopic dermatitis (AD), in which IL-13 signaling plays a dominant role. Keratinocytes express two receptors: The heterodimeric IL-4Rα/IL-13Rα1 and IL-13Rα2. The former one transduces a functional IL-13 signal, whereas the latter IL-13Rα2 works as a nonfunctional decoy receptor. To examine whether scratch injury affects the expression of IL-4Rα, IL-13Rα1, and IL-13Rα2, we scratched confluent keratinocyte sheets and examined the expression of three IL-13 receptors using quantitative real-time PCR (qRT-PCR) and immunofluorescence techniques. Scratch injuries significantly upregulated the expression of *IL13RA2* in a scratch line number-dependent manner. Scratch-induced *IL13RA2* upregulation was synergistically enhanced in the simultaneous presence of IL-13. In contrast, scratch injuries did not alter the expression of *IL4R* and *IL13RA1*, even in the presence of IL-13. Scratch-induced *IL13RA2* expression was dependent on ERK1/2 and p38 MAPK signals. The expression of IL-13Rα2 protein was indeed augmented in the scratch edge area and was also overexpressed in lichenified lesional AD skin. IL-13 inhibited the expression of involucrin, an important epidermal terminal differentiation molecule. IL-13-mediated downregulation of involucrin was attenuated in IL-13Rα2-overexpressed keratinocytes, confirming the decoy function of IL-13Rα2. Our findings indicate that scratching upregulates the expression of the IL-13 decoy receptor IL-13Rα2 and counteracts IL-13 signaling.

## 1. Introduction

Atopic dermatitis (AD) is a common, chronic or chronically relapsing, severely pruritic, eczematous skin disease that markedly deteriorates the quality of life of afflicted patients [1,2,3,4]. Lifetime prevalence of AD is estimated to be as high as 20% in the general population [5,6]. Clinical symptoms and signs of AD are characterized by skin inflammation, barrier dysfunction (xerosis), and itching [1,7]. Severe and chronic pruritus induces unavoidable scratching, and the vicious itch–scratch cycle exacerbates and perpetuates atopic inflammation and skin barrier function [8,9].

Compounding evidence shows that acute AD lesions have a significantly greater number of T helper 2 (TH2) cells expressing interleukin-4 (IL-4) and IL-13 than normal skin or uninvolved AD skin [10]. The TH2-deviated immune response is demonstrated both in pediatric and adult AD [11,12] and is greater in chronic than in acute lesions [11,13]. IL-4 and IL-13 inhibit filaggrin (FLG) and involucrin (IVL) expression in keratinocytes, leading to deteriorated barrier function [14,15]. IL-4 and IL-13 also potentiate the neuronal pruritic signal [16]. The pathogenic importance of IL-4/IL-13 signaling in AD has been recently highlighted because its blockage by dupilumab, a specific anti-IL-4 receptor α (IL-4Rα, *IL4R*) antibody, successfully improves skin inflammation in patients with AD [17]. Notably, a more recent large-scale transcriptomic analysis revealed a specific and dominant role of IL-13 in lesional AD skin, but nearly undetectable IL-4 expression was found [18]. 

The IL-13 signal is regulated via a complex receptor system. In nonhematopoietic cells, IL-13 engages a heterodimeric receptor composed of IL-4Rα and IL-13Rα1 (*IL13RA1*) [19,20]. IL-13Rα1 binds IL-13 with low affinity; however, when it forms a complex with IL-4α, it binds with much higher affinity, inducing the effector functions of IL-13 [19,20]. A second receptor, IL-13Rα2 (*IL13RA2*), is closely related to IL-13Rα1. IL-13Rα2 binds IL13 with high affinity, but it lacks any significant cytoplasmic domain and does not function as a signal mediator [20]. Cells with high IL-13Rα2 expression can rapidly and efficiently deplete extracellular IL-13 [21]. Likewise, IL-13 responses are enhanced in mice lacking *IL13RA2* [22]. These studies have highlighted that IL-13Rα2 can act as a scavenger or decoy receptor of IL-13 and elicits antagonistic activity against IL-13 [20].

Epidermal keratinocytes express IL-4Rα, IL-13Rα1, and IL-13Rα2 [23,24]. However, it remains unknown whether mechanical scratching affects the expression of these three IL-13 receptors. In this study, confluent keratinocyte sheets were scratched and the expression of IL-4Rα, IL-13Rα1, and IL-13Rα2 was assessed. Unexpectedly, this in vitro scratch model showed that scratch injuries upregulated IL-13Rα2 expression in a scratch line number-dependent fashion. This is the first report that scratch injuries may be able to produce an antagonistic signal against IL-13 by upregulating IL-13Rα2 expression.

## 2. Results

### 2.1. Scratching Upregulates the Expression of IL13RA2, Which is Further Enhanced by IL-13

**Figure 1:**
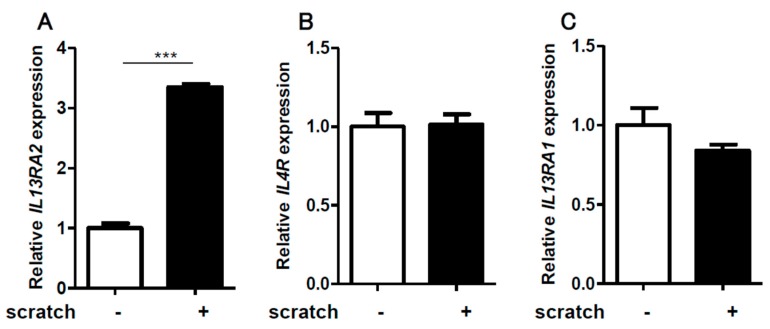
Scratching significantly upregulates the expression of *IL13RA2* in NHEK cells. A confluent keratinocyte culture was scratched with 14 lines, and the expression of *IL13RA2*, *IL4R*, and *IL13RA1* was analyzed by qRT-PCR and normalized to that of β-actin. Scratching significantly increased *IL13RA2* expression in NHEK cells (**A**). *IL4R* (**B**) and *IL13RA1* (**C**) expression was not altered. The cells were incubated for 6 h after scratching. Data is shown as the mean ± SEM (*n* = 3). *** *p* < 0.001.

**Figure 2:**
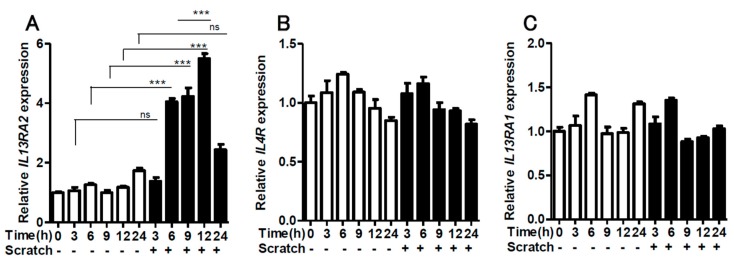
Time-course study for *IL13RA2* (**A**), *IL4R* (**B**), and *IL13RA1* (**C**) expression. The gene expression of *IL13RA2*, *IL4R*, and *IL13RA1* was measured with or without scratching at 0, 3, 6, 9, 12, and 24 h (*n* = 3). Data is shown as the mean ± SEM. ns: not significant. *** *p* < 0.001.

**Figure 3:**
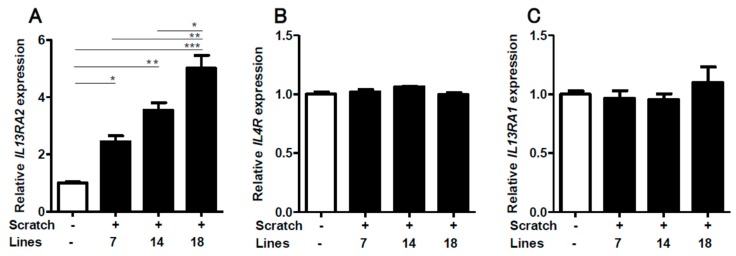
Scratching upregulated *IL13RA2* expression in a scratch line number-dependent manner. The keratinocyte sheet was scratched with 7, 14, and 18 scratch lines, and the gene expression of *IL13RA2* (**A**), *IL4R* (**B**), and *IL13RA1* (**C**) was measured (*n* = 3) 6 h after scratching. Data is shown as the mean ± SEM. * *p* < 0.05, ** *p* < 0.01, *** *p* < 0.001.

**Figure 4:**
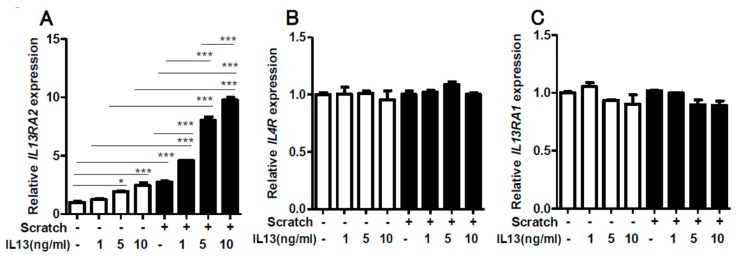
The effect of IL-13 on scratch-induced *IL13RA2* (**A**), *IL4R* (**B**), and *IL13RA1* (**C**) expression. Confluent keratinocyte sheets were non-scratched or scratched with 18 lines in the presence or absence of graded IL-13 concentrations (1, 5, 10 ng/mL). Cells were treated with IL-13 for 14 h before scratching and then incubated for another 6 h. Data is shown as the mean ± SEM. * *p* < 0.05, *** *p* < 0.001.

We first scratched confluent keratinocyte sheets in six-well culture plates with 14 scratch lines. The expression of *IL13RA2* was significantly enhanced in the scratched sheet, compared to that in the non-scratched control (Figure 1A). Notably, the gene expression of *IL4R* (Figure 1B) and *IL13RA1* (Figure 1C) was not affected by the scratch injury. The upregulation of *IL13RA2* gene expression was transient, peaking at 12 h and returning to a baseline level at 24 h (Figure 2A). The gene expression of *IL4R* (Figure 2B) and *IL13RA1* (Figure 2C) exhibited no differences over time. We next scratched the keratinocyte sheets with 7, 14, or 18 scratch lines. *IL13RA2* gene expression was significantly upregulated in a scratch line number-dependent fashion (Figure 3A). Again, *IL4R* (Figure 3B) and *IL13RA1* (Figure 3C) gene expression levels were not altered, irrespective of scratch line numbers. We next examined whether the simultaneous presence of exogenous IL-13 affected scratch-induced *IL13RA2* gene upregulation. Exogenous IL-13 itself significantly upregulated the baseline level of *IL13RA2* gene expression in non-scratched keratinocytes (Figure 4A). Notably, scratch-induced *IL13RA2* gene upregulation was significantly augmented synergistically by IL-13 in a concentration-dependent manner (Figure 4A). As shown in Figure 4B,C, graded concentrations of IL-13 did not alter the gene expression of *IL4R* and *IL13RA1* either alone or with a scratch injury.

### 2.2. Upregulation of IL-13Rα2 Protein in a Scratched Edge Area In Vitro as well as in Lesional AD Skin 

**Figure 5:**
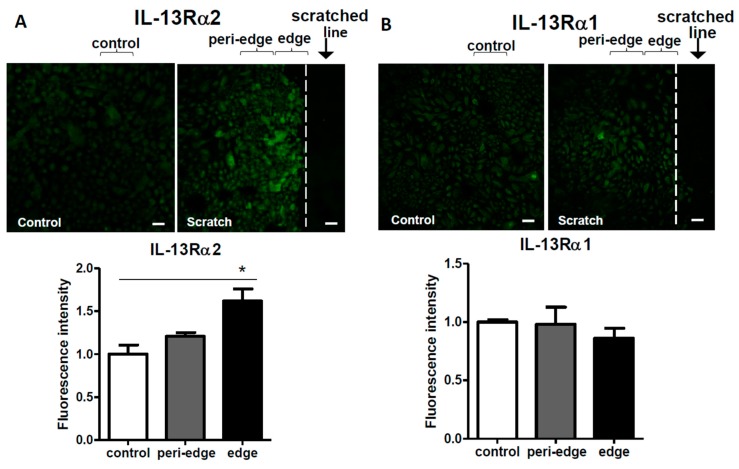
Immunofluorescence analysis for IL-13Rα2 (**A**) and IL-13Rα1 (**B**) proteins. Non-scratched control or scratched confluent keratinocyte sheets were immunostained with anti-IL-13Rα2 or anti-IL-13Rα1 antibodies. Data is shown as the mean ± SEM. * *p* < 0.05. Scale bar: 50 µm.

In order to determine the spatial expression of IL-13Rα2 protein in the scratched sheet, we conducted immunostaining. Immunostaining for IL-13Rα1 served as the unaffected control. The immunofluorescence intensity for IL-13Rα2 protein was significantly upregulated in the scratch edge area, compared to that in the non-scratched control (Figure 5A). The IL-13Rα2-positive signal was also slightly enhanced in the peri-edge area, but it did not reach statistical significance, compared to that in the non-scratched control (Figure 5A). In contrast, the immunofluorescence intensity for IL-13Rα1 protein was comparable among the scratch edge area, peri-edge area, and non-scratched control (Figure 5B). As a suitable anti-IL-13Rα2 antibody was unavailable, we were unable to detect IL-13Rα2 protein with western blotting. 

**Figure 6:**
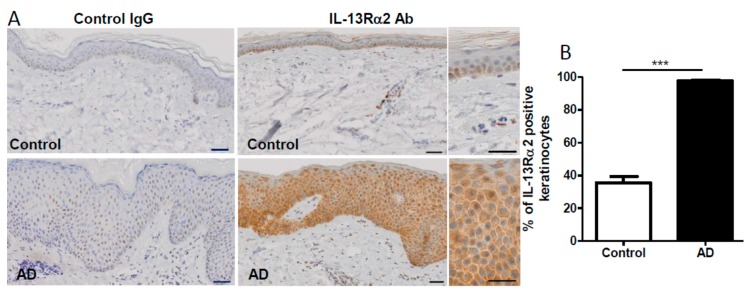
Immunohistochemical analysis for IL-13Rα2 expression. Control normal skin and lichenified lesional AD skin were immunostained with control IgG and anti-IL-13Rα2 antibody (**A**). The percentage of IL-13Rα2 positive keratinocytes was calculated in 11 normal skin and 11 AD skin samples (**B**). Data is shown as the mean ± SEM. *** *p* < 0.001. Scale bar: 50 µm.

We next immunostained IL-13Rα2 protein in lichenified lesional AD skin (*n* = 11) and normal control skin (*n* = 11). In normal skin, IL-13Rα2 expression was immunodetectable, especially in the epidermal basal layer (Figure 6A). Its expression was augmented in the lesional AD epidermis, compared to that in the normal control epidermis (Figure 6A). The percentage of IL-13Rα2-positive keratinocytes was significantly increased in the lichenified AD skin, compared to that in the normal control skin (Figure 6B).

### 2.3. Contribution of ERK1/2 and P38MAPK Activation to Scratch-Induced IL13RA2 Upregulation 

**Figure 7:**
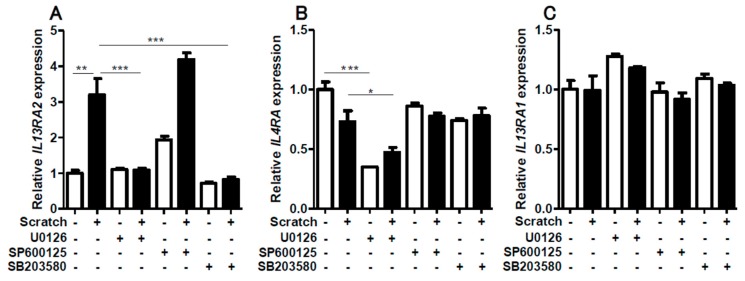
The effect of MAPK inhibitors on *IL13RA2* (**A**), *IL4R* (**B**), and *IL13RA1* (**C**) expression. Non-scratched and scratched confluent keratinocytes were treated with or without U0126 (MEK 1/2-ERK1/2 inhibitor), SP600125 (JNK inhibitor), and SB203580 (p38MAPK inhibitor). Data is shown as the mean ± SEM. * *p* < 0.05, ** *p* < 0.01, *** *p* < 0.001.

We next examined the MAPK signal transduction pathways leading to scratch-induced *IL13RA2* upregulation. The scratch injury upregulated the phosphorylation of ERK1/2, JNK, and p38MAPK (Supplementary Figure S1). Correspondingly, scratch-induced *IL13RA2* upregulation was disrupted in the presence of U0126 (MEK/ERK inhibitor) and SB203580 (p38MAPK inhibitor) (Figure 7A). Interestingly, SP600125 (JNK inhibitor) did not affect scratch-induced *IL13RA2* upregulation (Figure 7A). Baseline *IL4R* expression was downregulated only by U0126 (Figure 7B). The gene expression of *IL13RA1* was stable, irrespective of these inhibitors (Figure 7C). These results suggested that ERK1/2 and p38MAPK were involved in scratch-induced *IL13RA2* upregulation. 

### 2.4. IL-13-Mediated IVL Downregulation is Restored in IL-13Rα2-Overexpressed HaCaT Keratinocytes 

**Figure 8:**
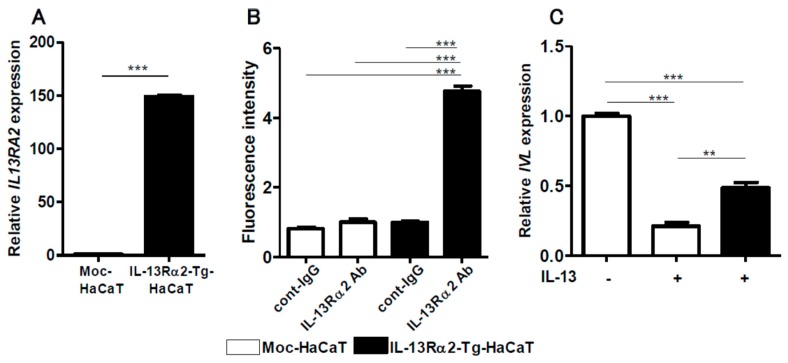
*IL13RA2* expression was upregulated in the IL-13Rα2-Tg-HaCaT cells more than in control Moc-HaCaT cells (**A**). Upregulated IL-13Rα2 protein expression was observed in the IL-13Rα2-Tg-HaCaT cells, compared to that in Moc-HaCaT cells (**B**). IL-13-induced *IVL* downregulation was partially restored in IL-13Rα2-Tg-HaCaT cells (**C**). ** *p* < 0.01, *** *p* < 0.001.

It is known that IL-13Rα2 exhibits a decoy function for IL-13 [20]. In order to examine this function, we established IL-13Rα2-Tg-HaCaT keratinocytes. The IL-13Rα2-Tg-HaCaT cells exhibited significantly higher expression of IL-13Rα2 mRNA (Figure 8A) and protein than the mock-HaCaT cells (Figure 8B and supplementary Figure S2). IL-13 is known to inhibit *IVL* and *FLG* expression in normal keratinocytes [14,15]. Likewise, IL-13 inhibited *IVL* expression in HaCaT keratinocytes (Figure 8C). However, IL-13-mediated IVL downregulation was partially, but significantly, attenuated in the IL-13Rα2-Tg-HaCaT keratinocytes (Figure 8C), suggesting that the decoy function of IL-13Rα2 was operative in keratinocytes.

Intriguingly, IL-13 downregulated *FLG* expression in normal human keratinocytes, but it failed to inhibit *FLG* expression in HaCaT keratinocytes (Supplementary Figure S3). Therefore, we did not focus further on *FLG* expression.

## 3. Discussion 

Itchiness is a specialized perception in the skin and an unpleasant sensation that elicits the desire to scratch in order to remove harmful stimuli, leading to a scratching behavior [25]. Scratching appears to exacerbate preexistent dermatitis in humans and mice [9,26], but it relieves the itching sensation [27]. Among cutaneous inflammatory skin diseases, the vicious itch–scratch cycle is particularly important in AD because it profoundly impairs the quality of life, treatment satisfaction and adherence, and socioeconomic stability of patients [28,29,30]. However, the subcellular biological effects caused by scratching keratinocytes remain elusive. As AD is a TH2-dominant, particularly IL-13-dominant, skin disease [18], we focused on whether a scratch injury affects the expression of three IL-13 receptors, IL-4R, IL-13Rα1, and IL-13Rα2, in keratinocytes.

In the present study, we demonstrated that the scratch injury enhanced the expression of an IL-13 decoy receptor, IL-13Rα2. Scratch-induced IL-13Rα2 upregulation was selective because no significant changes were recognized in the expression of the functional heterodimeric IL-13 receptor, IL-4R, or IL-13Rα1. Scratch-induced IL-13Rα2 upregulation was highly dependent on scratch stress because it was enhanced with more scratch lines. Moreover, immunofluorescence analysis revealed that the upregulation of IL-13Rα2 was largely confined to the scratch edge area where scratch stress was most observed. IL-13 itself enhanced IL-13Rα2 expression in keratinocytes, but this was less potent than with the scratch injury. However, strong and synergistic upregulation of IL-13Rα2 expression was observed with co-treatment of IL-13 and a scratch injury. 

Historically, the in vitro scratch injury of a keratinocyte sheet has been used as a good model for wound closure in that it reflects the migratory and proliferative capacity of keratinocytes [31,32,33]. Therefore, no previous studies have sought to examine the scratch-mediated alteration of IL-13 receptors. The selective upregulation of IL-13Rα2 was a novel and unexpected finding. We then investigated signal transduction that led to scratch-induced IL-13Rα2 upregulation. In our experimental model, the scratch injury augmented the phosphorylation of ERK1/2, JNK, and P38 MAPK. Likewise, inhibitors for ERK1/2 and P38 MAPK, but not JNK, disrupted scratch-induced IL-13Rα2 upregulation. These results suggest a crucial role of ERK1/2 and P38 MAPK in regulating scratch-induced IL-13Rα2 upregulation.

The vicious itch–scratch cycle is one of the cardinal features of AD [9,26]. Therefore, we examined epidermal IL-13Rα2 expression in lichenified (scratched) AD lesions. IL-13Rα2 expression was significantly increased in lesional AD skin, compared to that in the normal control epidermis. To determine the functional implications of IL-13Rα2 overexpression, we finally examined whether increased IL-13Rα2 expression suppresses an IL-13-mediated event, namely, IL-13-induced *IVL* downregulation [14,15,34]. As expected, IL-13 inhibited *IVL* expression, which was significantly restored in the IL-13Rα2 overexpressed keratinocytes. Based on these results, we deduced that scratch-induced IL-13Rα2 overexpression is biologically functional and may diminish IL-13-mediated hazardous events in the epidermal inflammatory microenvironment. 

Scratching may induce various biological consequences. The scratch signal exacerbates skin inflammation and conversely upregulates IL-13Rα2 expression, which may suppress excess IL-13 activity caused by the decoy function of IL-13Rα2 in keratinocytes. These fine-tuned mutually counteracting molecular events may participate in the formation of scratch-induced lichenified skin lesions.

## 4. Materials and Methods 

### 4.1. Reagents and Antibodies

Recombinant human IL-13 (Peprotech, Rocky Hill, NJ, USA) was dissolved in distilled water and added to the culture medium at a final concentration of 1, 5, or 10 ng/mL. The antibodies for immunofluorescence and immunohistochemistry staining were used as follows: Anti-IL-13Rα2 mouse monoclonal antibody (Abcam, Cambridge, UK), normal mouse IgG (Santa Cruz Biotechnology, Dallas, TX, USA), and goat anti-mouse IgG conjugated with Alexa Fluor 488 dye (Thermo Fisher Scientific, Waltham, MA, USA). The antibodies for western blotting were used as follows: Anti-ERK1/2, JNK, p38 MAPK, phospho-ERK1/2 (Thr202/Tyr204), phospho-JNK (Thr183/Tyr185), and phospho-p38 MAPK (Thr180/Tyr182) rabbit monoclonal antibodies and β-actin mouse monoclonal antibody (Cell Signaling Technology, Danvers, MA, USA) as primary antibodies, and anti-mouse IgG and anti-rabbit IgG HRP-linked antibody (Cell Signaling Technology) as secondary antibodies. Signal transduction inhibitor U0126 (ERK1/2 inhibitor) was purchased from Cell Signaling Technology. SP600125 (JNK inhibitor) and SB203580 (p38 inhibitor) were obtained from Tocris Bioscience (Bristol, UK). 

### 4.2. Cell Culture

Neonatal normal human epidermal keratinocytes (NHEKs) were purchased from Lonza (Basel, Switzerland) and cultured in KGM-Gold (Lonza), supplemented with bovine pituitary extract, recombinant human epidermal growth factor, insulin, hydrocortisone, gentamycin–amphotericin, transferrin, and epinephrine (Lonza) at 37 °C in 5% CO_2_. The medium was changed every 2 days. The cells reached 70–80% confluence and were passaged three times. The third passage of cells was used in all experiments. HaCaT cells (human keratinocyte cell line) were maintained in DMEM, supplemented with 10% fetal bovine serum (FBS) and antibiotics. Cells were passaged at 70–80% confluence and used in the experiment of transfection of plasmids.

### 4.3. In Vitro Scratched Keratinocyte Model

To establish the in vitro scratched keratinocyte model, NHEKs were seeded into 6-well plates (Corning, NY, USA) (3.5 × 10^5^ cells/well). Entire confluent keratinocyte sheets were scratched with 7, 14, and 18 lines, using 1000-μL tips (Greiner Bio-One, Kremsmünster, Austria), and incubated for 0, 3, 6, 9, 12, or 24 h at 37 °C in 5% CO_2_ after scratching. In several assays, the scratched cell sheets were treated with IL-13 (1, 5, or 10 ng/mL, Peprotech). 

### 4.4. Quantitative Real-Time PCR (qRT-PCR)

Total RNA was extracted from cells using RNeasy Mini Kit (Qiagen, Hilden, Germany), and cDNA was synthesized using PrimeScript RT Reagent Kit (Takara Bio, Shiga, Japan).

qRT-PCR was performed on the CFX Connect Real-Time PCR Detection System (Bio-Rad, Hercules, CA, USA), using TB Green Premix Ex Taq (Takara Bio). Denaturation was set at 95 °C for 30 s with 40 total cycles with a second step at 95 °C for 5 s. Annealing occurred at 63 °C for 30 s for *IL4R* and *IL13RA1*, and at 60 °C for 30 s for *IL13RA2* and *IVL*. The relative expression levels of *IL4R*, *IL13RA1*, *IL13RA2*, *IVL*, and *FLG* were normalized to that of β-actin. 

Gene-specific primers were as follows: *IL4R* forward, 5′-CTGCTCATGGATGACGTGGT-3′; reverse, 5′-CTGGGTTTCACATGCTCGCT-3′; *IL13RA1* forward, 5′-GTCCCAGTGTAGCACCAATGA-3′; reverse, 5′-GCTCAGGTTGTGCCAAATGC-3′; *IL13RA2* forward, 5′-GCTGGGAAGGTGAAGACCTA-3′; reverse, 5′-ACGCAAAAGCAGACCGGTTA-3′; *IVL* forward, 5′-TAACCACCCGCAGTGTCCAG-3′; reverse, 5′-ACAGATGAGACGGGCCACCTA-3′; *FLG* forward, 5′-TAACCACCCGCAGTGTCCAG-3′; reverse, 5′-ACAGATGAGACGGGCCACCTA-3′; β-actin forward, 5′-ATTGCCGACAGGATGCAGA-3′; reverse, 5′-GAGTACTTGCGCTCAGGAGGA-3′.

### 4.5. Immunofluorescence Analysis

Immunofluorescent analysis was performed on cell sheets cultured in 4-well slide chambers (Lab-Tek, Rochester, NY, USA) with KGM-Gold (Lonza) for 48 h, scratched using 1000-μL tips (Greiner Bio-One), and incubated for 6 h at 37 °C in 5% CO_2_. The cells were washed with phosphate-buffered saline 3 times for 5 min each and fixed in cold acetone for 10 min at room temperature. The cell sheets were blocked with 10% bovine serum albumin (Roche Diagnostics, Basel, Switzerland) and incubated with mouse monoclonal anti-IL13Rα2 (Abcam) or control normal mouse IgG (Santa Cruz Biotechnology). Goat anti-mouse IgG conjugated with Alexa Fluor 488 dye (Thermo Fisher Scientific) was used for the secondary antibody. The nucleus was stained with 4′,6-diamino-2-phenylindole (DAPI). Slides were then mounted with Ultra Cruz mounting medium (Santa Cruz Biotechnology) and were observed using a D-Eclipse confocal laser scanning microscope (Nikon, Tokyo, Japan). The immunofluorescence intensity was measured using ImageJ software.

### 4.6. Immunohistochemistry

Eleven lichenified lesional AD skin and 11 normal skin samples were embedded in paraffin by the conventional method and cut into 3-μm-thick sections. Antigen retrieval was performed using Heat Processor Solution pH 6 (Nichirei Biosciences, Tokyo, Japan) at 100 °C for 40 min, and endogenous peroxidase was blocked by incubating the sections with 3% H_2_O_2_ (Nichirei Biosciences). The sections were then incubated with anti-IL-13Rα2 (Abcam, 750×) antibody or control normal mouse IgG (Santa Cruz Biotechnology) for 30 min, followed by incubation with the secondary antibody, *N*-Histofine Simple Stain MAX-PO MULTI (Nichirei Biosciences). Immunodetection was conducted with 3,3-diaminobenzidine as the chromogen, followed by light counterstaining with hematoxylin. The number of IL-13Rα2-positive keratinocytes was counted in three high-power view areas, and the average percent positivity was calculated in each slide. 

### 4.7. Western Blotting 

Scratched or non-scratched cells were solubilized in complete Lysis-M (Roche Diagnostics, Rotkreuz, Switzerland). The cell lysates were prepared according to the standard protocol for western blotting analysis. The cell lysates were centrifuged at 14,000 rpm for 25 min and the obtained supernatants were used for analysis. The protein concentration was determined with a BCA protein assay kit (Thermo Fisher Scientific). Equal 20 μg amounts of protein were mixed with 4× LDS sample buffer (Invitrogen) and 10× sample reducing agent (Invitrogen), boiled at 70 °C for 10 min, loaded onto Bolt 4–12% Bis-Tris Plus (Thermo Fisher Scientific), and electrophoresed using Power Station III (Atto corporation, Tokyo, Japan) at 200 V and 180 mA for 25 min. The proteins were then transferred to an Immobilon PVDF Transfer Membrane (Merck, Kenilworth, NJ, USA), using Power Station III at 30 V for 1 h. Membranes were blocked with a blocking buffer, containing blocker diluent A and B, (Invitrogen) for 30 min. Membranes were probed overnight at 4 °C with the following primary antibodies: β-actin (Cell Signaling Technology), ERK1/2, JNK, p38, Phospho-ERK1/2, Phospho-JNK, and Phospho-p38 (Cell Signaling Technology). 

The secondary antibodies, anti-mouse IgG HRP-linked antibody (Cell Signaling Technology) and anti-rabbit IgG HRP-linked antibody (Cell Signaling Technology), were applied at room temperature for 30 min. Visualization of protein bands was accomplished with Super Signal West Pico Chemiluminescent Substrate (Thermo Fisher Scientific), using the ChemiDoc Touch Imaging System (Bio-Rad). 

### 4.8. Plasmid DNA and Transfection of Plasmids

Plasmids pCMV6-Entry (Mock) and *IL13RA2* (Myc-DDK-tagged), which contains a cytomegalovirus promoter, and the *IL13RA2* (NM_000640) human cDNA open reading frame clone were obtained from Origene Technologies (Rockville, MD, USA). The plasmids (1 µg) were dissolved in Amaxa P3 Primary Cell 4D-Nucleofector X Kit and were transfected into HaCaT cells using 4D-Nucleofector (Lonza, Basel, Switzerland), according to the manufacturer’s protocol. Transfected cells were then selected in Dulbecco’s Modified Eagle’s Medium (DMEM, Sigma–Aldrich, St. Louis, MO, USA) with 5% fetal bovine serum, Modified Eagle’s Medium Non-Essential Amino Acids, 10 mM HEPES, 1 mM sodium pyruvate (Thermo Fisher Scientific), and G418 disulfate aqueous solution (1500 µg/mL, Nacalai Tesque, Kyoto, Japan) for 3 weeks to obtain a stable cell line. 

### 4.9. Statistical Analysis

All data are presented as mean ± standard error of the mean (SEM). The significance of differences between groups was assessed using Student’s unpaired two-tailed *t*-test (two groups) or one-way ANOVA, followed by Bonferroni’s multiple comparison test (multiple groups), using GraphPad PRISM 5 software Version 5.02 (GraphPad Software, La Jolla, CA). *A p*-value of less than 0.05 was considered statistically significant.

## Supplementary Materials

Supplementary materials can be found at https://www.mdpi.com/1422-0067/20/13/3324/s1.

## Abbreviations

AD	atopic dermatitis
ERK	extracellular signal-regulated kinase
FLG	filaggrin
IL	interleukin
IL-4Rα	IL-4 receptor α
IL-13Rα1	IL-13 receptor α1
IL-13Rα2	IL-13 receptor α2
IVL	involucrin
JNK	c-Jun N-terminal kinase
MAPK	mitogen-activated protein kinase
MEK	mitogen-activated protein kinase kinase
NHEK	normal human epidermal keratinocyte
TH2 cell	T helper 2 cells
