# Supplementary material for: Scratching Counteracts IL-13 Signaling by Upregulating the Decoy Receptor IL-13Rα2 in Keratinocytes
Source: Int J Mol Sci. 2019 Jul 6;20(13):3324. doi: 10.3390/ijms20133324 (PMC6651282; doi:10.3390/ijms20133324)
Supplement: Supplementary file 1 [file ijms-20-03324-s001.pdf]

Suppl. Materials

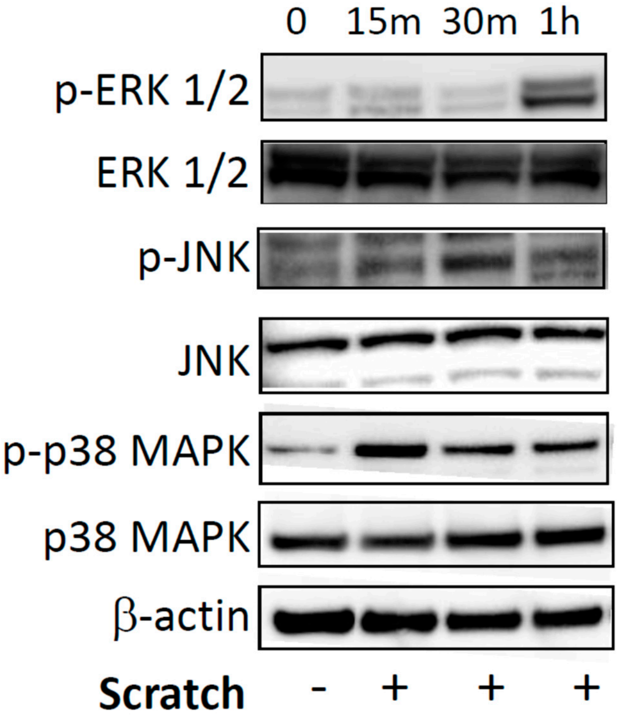

Figure S1. Confluent keratinocyte sheets were scratched with 18 scratch lines. Cellular proteins were obtained after 15 m, 30 m and 1 h after scratching. Phosphorylated and total amounts of ERK1/2, JNK and p38 were assessed by Western blotting. Amounts of β-actin were used for normalization.

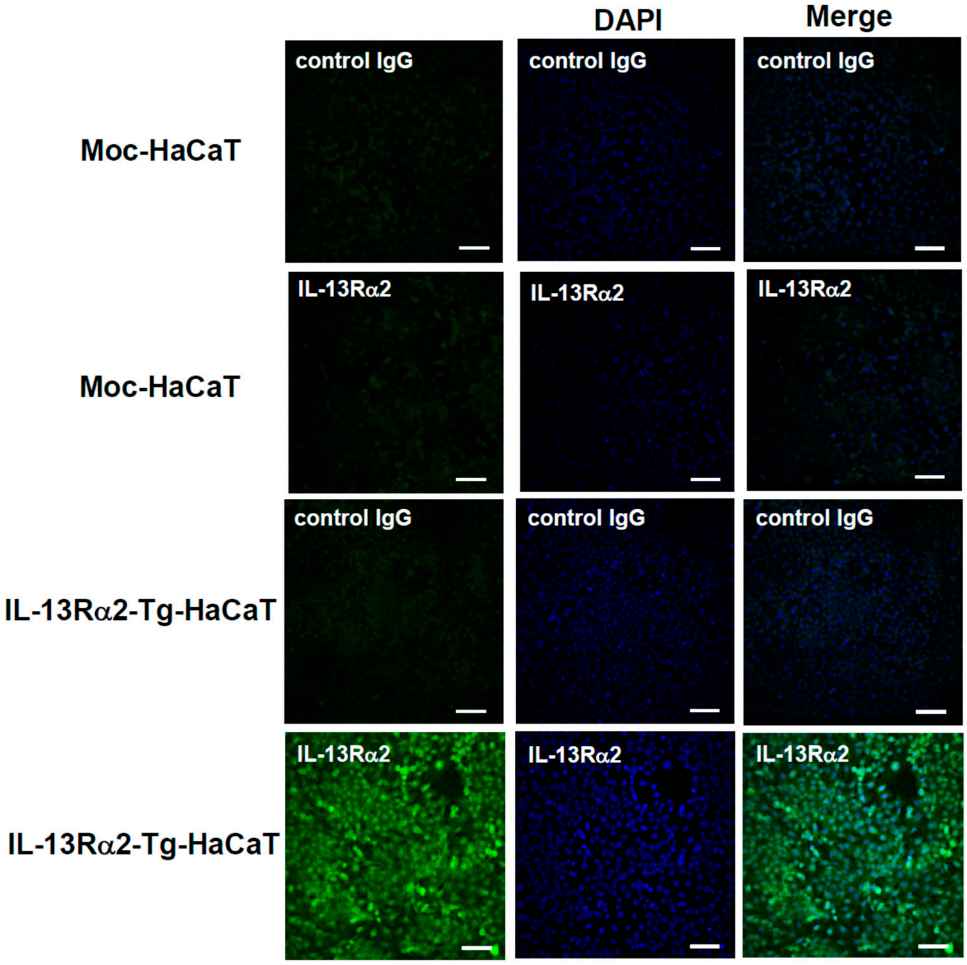

Figure S2. The protein expression of IL-13Rα2 was visualized in Moc-HaCaT and IL-13Rα2-Tg-HaCaT cells. Nuclei were stained with DAPI. Scale bar: 50 um.

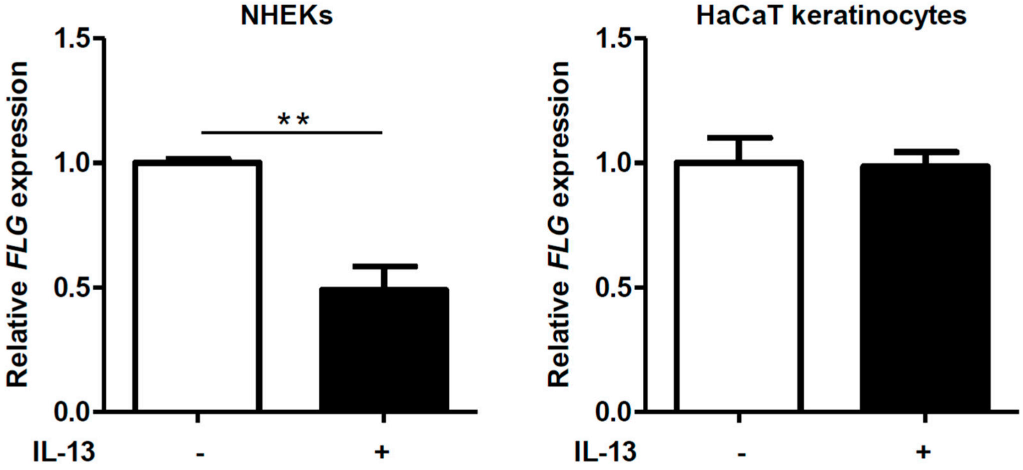

Figure S3. IL-13 inhibited the *FLG* expression in NHEKs but not in HaCaT keratinocytes. **: $p < 0.01$.
